# A New Embedded Key–Value Store for NVM Device Simulator

**DOI:** 10.3390/mi11121075

**Published:** 2020-12-02

**Authors:** Tao Cai, Qingjian He, Dejiao Niu, Fuli Chen, Jie Wang, Lei Li

**Affiliations:** 1 The School of Computer Science, Communication Engineering of Jiangsu University, Zhenjiang 212013, China; 13027520312@163.com (F.C.); wangjie287@live.com (J.W.); lilei@ujs.edu.cn (L.L.); 2 Jiangsu University Jingjiang College, Zhenjiang 212010, China; he_qingjian@outlook.com

**Keywords:** key–value storage system, key–value pairs management, I/O software stack

## Abstract

The non-volatile memory (NVM) device is a useful way to solve the memory wall in computers. However, the current I/O software stack in operating systems becomes a performance bottleneck for applications based on NVM devices, especially for key–value stores. We analyzed the characteristics of key–value stores and NVM devices and designed a new embedded key–value store for an NVM device simulator named PMEKV. The embedded processor in NVM devices was used to manage key–value pairs to reduce the data transfer between NVM devices and key–value applications. Meanwhile, it also cut down the data copy between the user space and the kernel space in the operating system to alleviate the I/O software stacks on the efficiency of key–value stores. The architecture, data layout, management strategy, new interface and log strategy of PMEKV are given. Finally, a prototype of PMEKV was implemented based on PMEM. We used YCSB to test and compare it with Redis, MongDB, and Memcache. Meanwhile, the Redis for PMEM named PMEM-Redis and PMEM-KV were also used to test and compared with PMEKV. The results show that PMEKV had the advantage of throughput and adaptability compared with the current key–value stores.

## 1. Introduction

The development of components in computer systems is unbalanced. The I/O performances of the disk and the Flash-based solid-state device (SSD) are much lower than the speed of the CPU. Nowadays, a series of NVM (non-volatile memory) devices was developed, such as phase-change memory (PCM) [1], spin-transfer torque random access memory (STT-RAM) [2], and Intel’s 3D X-point [3], etc. These devices have the advantage of byte-addressable, longer writing life compared with Flash, lower power consumption and close I/O speed of dynamic random access memory (DRAM). Hence, the NVM device is an important way to solve the memory wall problem in computer systems. However, the current I/O software stack was designed for low-speed storage devices such as disks and flash-based SSD, which hinders the performance of the NVM storage system and its application. Relevant research has found that the I/O software stack accounts for more than 94% of the time overhead in NVM storage systems [4]. Meanwhile, applications generally access the storage system by the file system. The current research shows that the latency of the file system accounts for 30% of the total delay and results in 85% I/O performance loss of the NVM storage system. Therefore, how to design new NVM storage systems according to the characteristics of the application is an important factor.

The key–value store is an important application, and it has been widely used in many information systems, such as Dynamo at Amazon [5], Voldemort at LinkedIn [6], Cassandra at Apache [7], LevelDB at Google [8], and RocksDB at Facebook [9]. It is tailored in many data-intensive Internet applications, such as social networking, e-commerce, and online gaming. Facebook has analyzed the key–value pairs in key–value store and discovered that more than 90% key–value pairs are smaller than 500 bytes [10]. At the same time, only 2 bytes are used for user account status in the key–value store of Facebook, and it increases the difficulty of key–value pairs management. Therefore, the key–value store needs to have high random I/O performance. Meanwhile, the operation of the key–value store is relatively simple, and it facilitates the design and implementation of the key–value store. The put operation is for writing data, get operation is for reading data and Delete operation is for deleting data in key–value store. However, the large number of key–value pairs is an important factor that restricts the I/O performance of key–value store. Current key–value stores need to access the storage device several times in order to search for a key–value pair. Due to the high reading and writing speed of NVM devices, the access interface becomes an important factor affecting the I/O performance of NVM devices. Therefore, several access times for the NVM device should be an important factor in the latency and throughput of key–value store NVM devices. At the same time, current key–value stores need to access NVM devices by the file systems and thus will cause the copy of index and key–value pairs between user space and kernel of OS. Meanwhile, these data are also needed to transfer and convert among several layers in the I/O software stack, such as system call, file system and block layer. There are several caches for accessing key–value pairs, such as page cache and device buffer. It will affect the latency and throughput of key–value store for the NVM devices. The structure of current NVM devices is similar to the Flash-based SSD. There is a built-in CPU with strong processing capabilities like ARM in NVM devices; many tasks can be dealt with efficiently by NVM devices itself and which also can be used to improve the management efficiency of key–value pairs. Therefore, designing a new key–value store for NVM devices is an urgent issue.

The main contributions of this article are as follows:

1. Different from the current I/O stack shorten method for NVM, the key–value storage engine is embedded in the NVM device. All key–value pairs are managed by the processor embedded in the NVM device, and it can reduce the data transfer between the NVM device and main memory.
2. The key and value index of key–value pairs is divided and managed in a different way, which reduces the size of the index and is suitable for the limited memory and processing capacity of NVM devices.
3. The new interface of NVM devices is designed to access the embedded key–value storage engine directly for the application. It can bypass the file system and block layer to improve the latency and throughput of key–value store via shortening the I/O software stack.
4. Different from the current index key in key–value pairs by the B+ tree, only the leaf nodes of the B+ tree are stored in the NVM device, which can reduce the capacity overhead of the NVM device and improve the index efficiency by DRAM.
5. A new consistency mechanism based on a two-layer log is designed to reduce the extra space and time overhead of the key–value store consistency.
6. The prototype of the embedded key–value store named PMEKV is implemented based on the open-source NVM device simulator named PMEM. The embedded key–value interface and embedded key–value storage engine are added to the current PMEM. Due to the lack of actual NVM devices that can be reprogrammed, the simulator of NVM devices is used. PMEM is just the device driver for Linux, so the current PMEKV cannot avoid the data transfer between NVM devices and host. However, It still can shorten the I/O software stack to prove the advantage of our design. We use YCSB to test and compare it with Redis, MongoDB and Memcached. The test results show that PMEKV has higher performance and better adaptability than current key–value stores.

## 2. Related Works

There is much research on how to improve the efficiency of storage systems based on NVM devices. A PCIe PCM array named Onyx was implemented and tested [11]. The results showed that it could improve the performance of reading and small writing by 72~120% compared with the Flash-based SSD. FusionIO extended the support of file systems for atomic writing [12]. It could convert write requests in MySQL to atomic write requests of NVM devices and improve the transaction efficiency by 70%. S. Kannan used NVM devices to store checkpoints locally and remotely [13]. The pre-replication policy was designed to move the checkpoint from memory to NVM devices. The efficiency of metadata management largely affects the I/O performance of a file system. In general, metadata are stored with the data of a file in blocks, and a small modification to the metadata leads to the update of the entire block. To take advantage of NVM devices to optimize the access performance of the metadata, PMFS is designed as a lightweight file system for NVDIMMs [14]. The size of the log unit is the same as cache lines to ensure file system consistency, which reduces the performance impaction of metadata updates. Youyou Lu designed blurred persistence for persistent transactional memory [15]. It could blur the volatility-persistence boundary to reduce the overhead of transactions and improve performance by 56.3% to 143.7%. Qingsong Wei proposed the persistence in-memory metadata management mechanism (PIMM), which reduces the SSD I/O traffic by utilizing persistence and byte-addressability characteristic of NVM devices [16]. PIMM separated data from the metadata access path and stored the data on the Flash-based SSD and the metadata on NVM devices.

There is also much research focusing on the overhead of the I/O software stack and the performance loss by accessing the interface of storage devices. Steven Swanson analyzed the hardware and software overhead of the storage system based on NVM devices [4] and found that the current I/O software stack needs to be reconstructed. The I/O software stack overhead of the NVM device with a traditional block interface is 18%, while PCIe-NVM is 63%. The overhead of the I/O software stack greatly affects the NVM device to increase bandwidth and reduces the latency of the storage system. The shortcomings of the traditional block interface and the new batch processes multiple I/O requests are proposed to achieve atomic writes of NVM devices, which can improve the efficiency of applications, file systems, and operating systems. Moreover, direct access to NVM devices is also very popular. DEVFS also reminded that the I/O software stack should be carefully redesigned for the characteristics of the storage device [17]. The application should be trapped in the kernel of the operating system and involved in many layers such as memory buffers, cache, file system and block layer using the traditional I/O software stack. They greatly increased the access latency and reduced the benefits of the NVM device with high I/O speed. Researchers remind that file systems also account for a large proportion of overhead for the I/O software stack, so it was important to optimize or current file systems. In the PCIe-NVM prototype system, the file system accounted for 30% of the total latency and reduced performance by 85%. Adrian Caulfield explored the interface optimization technology for SCM [18], and the hardware access control was proposed to avoid the time overhead of context switch between kernel space and user space when accessing the file system. Meanwhile, the functions of the file system were embedded in the application to improve flexibility. The POSIX also affected the efficiency of the storage system based on NVM devices. The direct I/O was used to access NVM devices directly without metadata modification and reduce the access control overhead of the file system. The hardware-based access control of the file system was used to separate access paths for metadata and data [19,20] and access storage devices directly in userspace. In addition, in order to take advantage of the byte-addressability of NVM devices, it is necessary to pay attention to the granularity of access and update. Haris Volosl designed a lightweight access interface for NVDIMMs to manage and ensure data consistency named Mnemosyne [21]. Jeremy Condit used the load/store instruction to access the NVDIMMs directly and improve efficiency [22].

Many studies were focused on how to improve the search efficiency and access performance of key–value pairs according to the characteristics of key–value store. The flexible and efficient key–value store is very useful in many applications, such as social networks. Xingbo Wu designed LSM-Trie [23]; it was a prefix tree to improve the efficiency of the key–value pair management. The limited writing lifetime is the shortcoming for NVM such as PCM. Therefore, there is much research on how to reduce the write times of NVM devices [24,25,26,27]. Shimin Chen proposed an unordered leaf node B+ tree to reduce the write caused by sorting [24]. Fei Xia optimized key–value operation by the hybrid storage system composed of NVM devices and other storage devices and hybrid index [28]. In order to take advantage of the byte-addressable nature of the NVM device, Deukyeon redesigned the B+ tree to ensure the consistency between NVM devices and the cache line. The PMEM-KV is an open-source key–value store for the NVM device simulator [29]. It implemented a persistent memory-aware queue of direct NVM devices access by the linked list and PMDK, which was the open-source persistent memory development kit from Intel. NVMKV optimized the key–value store based on the characteristic of the NVM device internal structure. A lightweight key–value store was implemented by sparse address space with FTL, dynamic mapping, and lock-free parallel mechanism [30]. The ratio of the get operation to set operation is up to 30:1 in the cache of the key–value store. This means that concurrency is very important to the storage system of the key–value store. The NVM device has high parallelism I/O performance. Echo [31] and NVStore [32] were two key–value stores for SCM with MVCC for concurrency control in the NVM device. Wentao Han [33] and Hyeontaek Lim [34] used partition to improve concurrency of hash tables. PALM is a lock-free concurrent B+ tree [35]. Hua Fan proposed a counterexample to prove that the concurrent transaction can reduce the time overhead by no confliction and designed the epoch-based ECC mechanism to minimize the overhead of synchronization confliction [36]. We also have designed the matrix key–value storage system to use several NVM devices and improve the efficiency of key–value management [37].

## 3. Challenges

**Figure 1:**
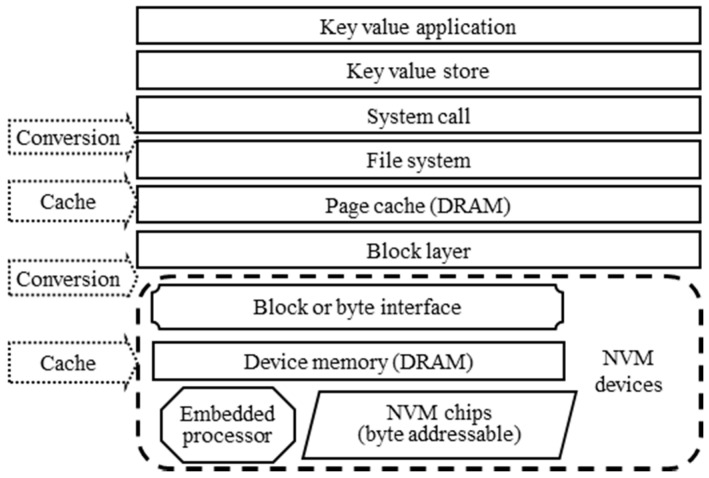
The structure of key–value store for the NVM devices.

The structure of the current key–value store for the NVM device simulator is shown in Figure 1. To access the key–value pair stored in the NVM device, the key–value application needs to go through multiple levels such as key–value store, file system, page cache, block layer, device interface and device cache. There are several conversions of data and operation and multiple caches built by DRAM. They seriously affect the latency and throughput of key–value store. In addition, the embedded processor in NVM devices has not been used in the current key–value pair management system. In general, the key–value application has the following challenges with NVM devices.

1. Low-speed interface. The NVM device has extremely high I/O performance and a relatively slow interface. Currently, the PCIe, SAS, and SATA are the main interfaces for the NVM device simulator with a large storage capacity. Their speed is low than NVM devices due to the limitation of the computer structure and hardware, and it is difficult to make an improvement in a short time.
2. Block interface. Currently, there are a large number of NVM devices with a block interface, which causes serious write amplification and affects I/O performance. For key–value stores, where the key–value pairs are generally small items and some just 1 KB, the problem is more prominent. NVDIMMs support byte-addressing, but the current key–value store is designed for a traditional storage device with a block interface. Therefore, the byte-addressing of NVM devices is also difficult to improve the latency and throughput of key–value store without any modification.
3. The unoptimized embedded processor. Similar to the Flash-based SSD, NVM devices are generally equipped with embedded processors. Due to the longer lifetime of NVM compared with Flash-based SSDs, the embedded processor should have more computing power when completing the self-management. However, the current key–value store lacks a corresponding optimization strategy and cannot use the embedded processor to deal with parts of the functions of key–value store, which also affects the I/O performance of the key–value store. As the interface of the NVM device is the bottleneck, the idea of a big data system can be used to move the key–value pair management to NVM devices, thus reducing the data access of NVM devices. It should be a useful way to improve the performance of key–value store for the NVM device simulator with a block interface.
4. The long I/O software stack. When reading and writing NVM devices, the key–value store needs to go through system calls, file systems, page caches and block layers. There are multiple data conversions and several data copies between user space and OS kernel. At the same time, there are several caches, such as page caches in the operating system and caches in NVM devices. The read and write speed of the NVM device is already close to the DRAM that built these caches. Therefore, these caches should affect the latency and throughput of key–value store. In addition, the current I/O software stack is designed and optimized for accessing a large amount of data, and it is difficult to effectively adapt to random read and write of small data for key–value pairs.

## 4. The Design of PMEKV

### 4.1. The Architecture of PMEKV

**Figure 2:**
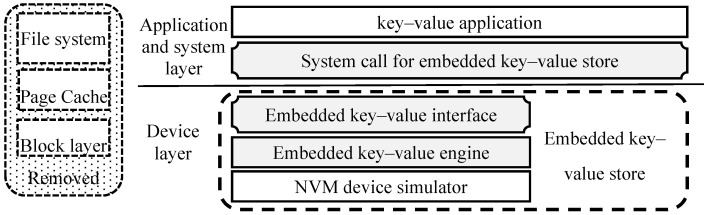
The structure of a new embedded key–value store for the NVM device simulator.

We modify the key–value store and the I/O software stack to design a new embedded key–value store for the NVM device simulator named PMEKV. The structure is shown in Figure 2.

We have analyzed the bottleneck of the current key–value system in Section 3. Compared with the current architecture shown in Figure 1, several layers in the current I/O software stack are bypassed, such as page cache, file system and block layer. This can shorten the I/O software stack and reduce the time overhead for accessing key–value pairs stored in NVM devices. Their functions should be replaced by the embedded key–value interface, embedded key–value storage engine and system calls for embedded key–value store. The embedded key–value interface and storage engine should be added to the NVM device and implement a new embedded key–value store for the NVM device simulator. These two modules can move the key–value management function from the application to the NVM device and reduce the data transfer between NVM devices and main memory. At the same time, the new system calls for embedded key–value stores that are implemented and exposed to key–value applications. They receive the access request submitted by the key–value application, and forward it to the embedded key–value store for the NVM device simulator and return the result. The embedded key–value interface is responsible for receiving the access requests and converting them into specific key–value operations. The embedded key–value storage engine is responsible for managing the key–value pair stored in the NVM device and completing the access request of key–value applications.

The search, access, and management of all key–value pairs are performed by NVM devices itself. It can reduce the access time of NVM devices and avoid the large transfer between NVM devices and main memory. Meanwhile, it alleviates the interface impaction of NVM devices to the latency and throughput of key–value store. Therefore, the embedded key–value storage engine in NVM devices can effectively utilize the byte-addressable of NVM devices to improve the efficiency of key–value pairs management and avoid write amplification. These systems call for embedded key–value stores that can be used to shorten the I/O software stack for a key–value application of accessing key–value pairs and reduce the time overhead.

However, there are some limitations of PMEKV with bypass several layers in the current I/O software stack. The first is that the current application needs to be reimplemented with the interface of PMEKV. The second is that the kernel of Linux needed to be modified and add some system call for PMEKV, it will give some troubles when OS Kernel update. The last, the OS cannot use the NVM device with PMEKV as the general storage device to store data again; if there is not some protection that the OS should destroy the data of PMEKV.

### 4.2. The Data Layout of PMEKV

In addition to the embedded processor, NVM devices are equipped with the device memory and the NVM chip. The storage capacity device memory is less compared with the NVM chip, but it does not have the limitation of the write lifetime and the read and write speed are also higher than NVM chips. When the B+ tree is used to index the key–value pairs, the storage size of the value in key–value pairs is more dynamic and larger than the key in it, which increases the consumption of device memory and affects the efficiency of key–value pairs management. To deal with it, we separate the key and value in key–value pairs. Then, the address of value stored in NVM chips named Value_Pointer is stored in the leaf node of the B+ tree instead of the value itself. We design a new structure of the lead node of the B+ tree named LeadNode. Therefore, it can optimize the device memory in NVM devices and improve the management efficiency of key–value pairs.

Struct LeadNode { char *Key[]; void *Value_pointer[];//address of Value in NVM int num;//the number of Key contained in the node struct InnerNode *parent;//point to the parent node struct LeadNode *next;//point to the next LeadNode }

**Figure 3:**
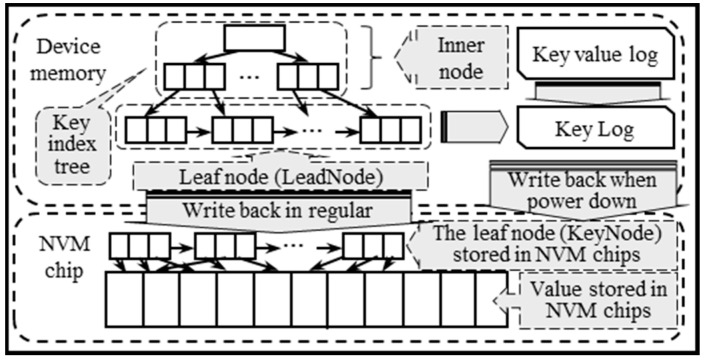
The data layout of PMEKV.

As shown in Figure 3, the B+ tree named key index tree is used to index all keys in the key–value pairs and stored in the device memory. The key–value log and the log of LeadNode named key log are also stored in the device memory, while the information of LeadNode is stored in the NVM chip to reconstruct the key index tree when the key–value store is restarted. Meanwhile, the value of the key–value pair is stored in the NVM chip.

We design a new structure named KeyNode to store the information of LeadNode in NVM chips. The structure is as follows: The two lists named key and Value_pointer should not contain the null value, and there are no values of the number of key and Value_pointer. Therefore, the size of KeyNode is less than LeadNode. The data that need to be written back or read from NVM chips can be reduced, especially when many values in the key and Value_pointer list are null. The speed of store key–value pairs and recover key–value store can be improved.

Struct KeyNode { char *Key[]; void *Value_pointer[];//address of value in NVM chips struct KeyNode *next;//point to the next KeyNode }

### 4.3. The Management of Key–Value Pair

The processing flows of the main operations in PMEKV are as follows:

The insert operation is used to insert a new key–value pair into PMEKV. The pseudo-code is as follows:

Insert (Key_New, Value_New): Inserting one key–value pair to PMEKV  Search the Key index in the device memory;  Find the LeadNode to be inserted Key_New;  if (this LeadNode is full) {   Splitting this LeadNode;   Find the LeadNode to be inserted; }  Write Value_New to the NVM device and obtain the address;  Insert Key_New and the address of Value_New in the NVM device into this LeadNode; Return the successful insert information.

The find operation is used to find the key–value pair corresponding to the key in PMEKV, and the pseudo-code is as follows:

Search (Key_Find, Value_Return): Searching one key–value pair in PMEKV by key  Search the key index in the device memory;  Find the LeadNode that Key_Find may appear;  If (exist the key same as Key_Find in LeadNode) {   Access the Value_Pointer corresponding to this key;   Access the data corresponding to this Value_Pointer in the NVM device;   Assign this data to the Value_Return and return it; }  Else   Return information that there is not the key value pair corresponding to Key_Find.

The delete operation is used to find and delete the key–value pair corresponding to the key in PMEKV. The pseudo-code is as follows:

Delete (Key_Delete): Deleting one key–value pair in PMEKV by key  Search the key index in the device memory;  Find the LeadNode that Key_Delete may appear;  If (exist the Key same as Key_Delete in LeadNode) {   Access the Value_Pointer corresponding to this Key;   Delete the data in the NVM device corresponding to this Value_pointer;   Delete the corresponding key and value pointer in this LeadNode;   Adjust the tree structure as needed;   Return information that the key value pairs is deleted successfully; }  Else   Return information that there is not the key value pair corresponding to Key_Delete.

The update operation is used to find the key–value pair corresponding to the key in PMEKV and update the corresponding value. The pseudo-code is as follows:

Update (Key_Update, Value_New): Updating one key–value pair in PMEKV  Search the key index in the device memory;  Find the LeadNode that Key_Update may appear;  If (exist the key same as Key_Update in LeadNode) {   Access the Value_Pointer corresponding to this key;   Replace the data in the NVM device corresponding to this Value_pointer with Value_New;   Return information that the key–value pairs are updated successfully; }  Else   Return information that there is not the key–value pair corresponding to Key_Update;

The interval traversal operation is used to traverse all key–value pairs whose keys are between Key_Start and Key_End in PMEKV. The pseudo-code is as follows:

Interval_traversal (Key_Start, Key_End): Traversing several key–value pairs in PMEKV  Search the key index tree in the device memory;  Find the LeadNode that Key_Start may appear;  Check whether the Key in the leaf node equals to or less than Key_Start;  If (exist the key >= Key_Start and Key < Key_End) {   Do {    Access the Value_pointer corresponding to this key;    Access the data corresponding to this Value_Pointer in the NVM device as value;  Return key–value pairs with this key and value;  If (exist the key unvisited in this LeadNode)     Move to the next key;    Else  Move to the first key in the next LeadNode;   }   While (Current key in this LeadNode <= Key_End)  }  Else   Return information that there is not the key–value pair with the key between Key_Start and Key_End;}

Although the key–value pairs are returned to key–value applications, all operation are done by the NVM device itself, which avoids frequent transfer of a large amount of data between NVM devices and key–value applications, and effectively improves the access and management efficiency of key–value pairs.

### 4.4. The Interface of PMEKV

When accessing the NVM device, there are many layers in the current I/O software stack, such as cache, file system, and block layer. This long I/O software stack greatly affects the read and write performance of NVM devices.

**Figure 4:**
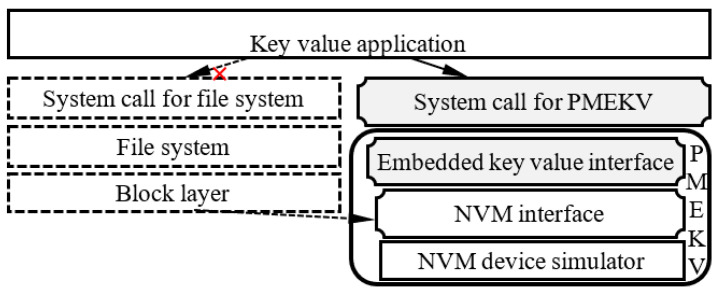
The interface of PMEKV.

As shown in Figure 4, a new interface is designed for PMEKV and key–value applications. The embedded key–value interface is implemented in PMEKV corresponding to those operations described in Section 4.3, and the new system calls for PMEKV are implemented in OS for key–value application. Therefore, the I/O software stack for key–value application can be shortened to improve the I/O performance of key–value store and ensure the NVM device deal with the key–value operations by itself to avoid the data transfer. At the same time, the modification of the current key–value application can be avoided by the system calls of PMEKV.

### 4.5. The Two-Tier Log

**Figure 5:**
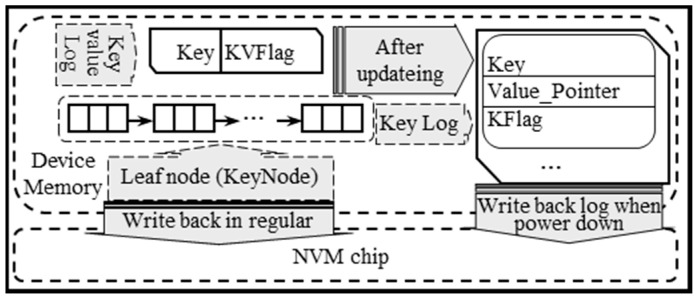
The schematic diagram of consistency strategy based on the two-tier log.

The data stored in the memory of NVM devices will be lost on power down and result in inconsistency of key–value store. A two-tier log is designed to ensure the consistency of PMEKV. It contains the key–value log and the key log, corresponding to the update of key–value pair and LeadNode in key–value tree, respectively. The schematic diagram is shown in Figure 5.

A tuple of (key, KVFlag) is used to represent each entry in the key–value log. Key corresponds to the value of key in the key–value pair that is needed to be updated. KVFlag indicates the status of the current log, with 0 indicates that the key–value pair was updated and stored on NVM chips, 1 indicates that the insertion of the key–value pair has not been stored on NVM chips, 2 indicates that the deletion of the key–value pair has not been done on NVM chips, and 3 indicates that the update of the key–value pair has not been done on NVM chips. Meanwhile, each key log entry can be represented by a triple of (key, Value_pointer, KFlag), where the key corresponds to the key in LeadNode that should be updated, Value_pointer is the address of the value of this key–value pair on NVM chips. KFlag is the status of the log. 0 indicates that the information of this LeadNode was written to the NVM chip, 1 indicates that the information of LeadNode with inserted key–value pairs is not written to the NVM chip, 2 indicates that the information of LeadNode with deleted key–value pairs is not written to the NVM chip, and 3 indicates that the information of LeadNode with updated key–value pairs is not written to the NVM chip.

Before PMEKV updates the key–value pair, the key of key–value pairs is written into the key–value log. Meanwhile, the value of key–value pair is written to the NVM chip, and the KVFlag in key–value log is set to 1, 2 or 3 according to the operation type. After the key index tree is modified, KVFlag is set to 0, and the operation of the key–value pair is finished. At the same time, the key and Value_pointer of the corresponding key–value pair in the key index tree is written to the key log, and KFlag is set to 1, 2 or 3 according to the operation type.

When the key–value store powers down, the entries with none zeros of KVFlag and KFlag in the key–value log and key log are written back to the NVM chip. When the key–value store restarts, the key–value log stored in NVM chips is used to perform the corresponding operation again, and the key log is used to update the KeyNode stored on NVM chips and rebuild the key index tree.

During the execution of PMEKV, the LeadNode in the key index tree should be periodically written back to NVM chips as the KeyNode does by the workload. Then, all KFlags in the key log are set to 0. Meanwhile, the entries should be periodically deleted from key–value pair log and key log once their KVFlag or Kflag is 0. The HTM is used to ensure the atomicity update of the key–value pair during insertion, deletion, and update.

A single key–value pair, key and Value_Pointer in the index can be accessed concurrently with the consistency mechanism based on a two-tier log, which can reduce the extra time overhead and also ensure the consistency of the key–value index tree and key index tree.

## 5. Evaluation

First, we implement the prototype of PMEKV, and YCSB is used to test its performance. We compare it with Redis, MongDB, and Memcache. At the same time, researchers have realized two key–value stores PMEM-KV [29] and PMEM-Redis [38]. The PMEM-KV and PMEM-Redis do not implement the key–value management engine in the PMEM; they use the same architecture in Figure 1 but only exploit PMEM to replace current storage devices such as HDD or SSD for key–value store and Redis. Moreover, PMEM-KV and PMEM-Redis still have the limitation due to the current long I/O software stack. We also compare them with PMEKV.

### 5.1. The Prototype

PMEM is a popular open-source NVM devices simulator from Intel; now, it is also the component of the Linux kernel [39,40,41]. It is also the device driver for the Intel Optane persistent memory [42,43], which is the actual NVM devices. We modify the source code of PMEM to implement the embedded key–value interface and the key–value storage engine and build up the prototype of PMEKV. Meanwhile, the system calls for PMEKV are implemented in Linux for key–value application, where the Linux kernel is modified to reserve 50G kernel space at the end of the kernel address for PMEKV and PMEKV is used as raw devices in Linux. The machine used for testing has two Intel E5 processors, 128 GB main memory and a 120 GB Flash-based SSD. The operating system is Centos7, and the version of the Linux kernel is 4.4.112.

In order to test and analyze PMEKV, we implement three prototypes, respectively. PMEKV-NVM has the embedded key–value storage engine into PMEM. PMEKV-M stores key index in main memory based on the B+ tree, and value of key–value pairs is also stored in main memory, which is an in-memory key–value store. PMEKV-FS stores key–value pairs in PMEM, while the key index tree is kept in main memory and maintained by CPU. Three popular key–value stores, Redis, MongoDB and Memcached, are selected for comparison. They are all be set in memory mode. YCSB is used as the testing tool. Five basic interfaces, such as read, insert, delete, update, and scan, are implemented. Meanwhile, YCSB is run as the local mode to avoid the impact of the network. There are two stages in YCSB, that is load and run. The key–value pairs should be inserted into the key–value store in the load stage, and the access to the key–value pairs should be done in the run stage. The two workloads in YCSB named Workloada and Workloadb, are used for testing. The Workloada is a cloud system benchmark from Yahoo, and it uploads a heavy workload. There are 1000 records and operations in Workloada, and the percent of read and update operations is 50%. The size of each record is 10 KB. The Workloadb is also a cloud system benchmark from Yahoo, but it is read mostly workload. The number of records and operations are the same as Workloada. The percent of the read operation is 95%, and the update operation is 5%. The size of each record is 10 KB.

### 5.2. Key–Value Pairs Insertion

At first, 1000 key–value pairs are inserted into key–value store for testing throughput, time overhead and the average delay in a single-threaded mode. The results are shown in Table 1.

As seen from Table 1, compared with PMEM-KV and PMEM-Redis, PMEKV-NVM improves the insert throughput by 5% and 4%, respectively, and the time overhead also can be decreased to verify that the PMEKV has the latency advantage. PMEKV-NVM can improve the insert throughput of key–value pairs and reduce the latency compared with Redis, MongoDB and Memcached. Specifically, compared with MongoDB, the throughput of PMEKV is increased by 2.5 times, and the time overhead and average delay are reduced by 71% and 65%, respectively. This is because that the Flash-based SSD is still used to make up for the insufficient capacity of main memory, although MongoDB is set as the in-memory mode. The key–value pairs need to be mapped into the memory when accessed by YCSB, which seriously affects the insertion throughput of MongoDB. Compared with Memcached, PMEKV-NVM can also increase throughput by 1.6 times, with time overhead, and average latency can be reduced by 60% and 58%. It indicates that PMEKV-NVM can reduce the read and write time of NVM devices as the embedded key–value storage engine is used to manage the key–value pairs. Thus, PMEKV-NVM achieves better performance than other in-memory key–value stores on key–value pairs insertion. Compared with Redis, PMEKV-NVM also increases the throughput by 6%, and the time overhead and average delay are reduced by 3% and 17%, respectively. This also shows that PMEKV-NVM can effectively improve insert throughput. Compared with PMEKV-FS, PMEKV-NVM has the performance advantage on key–value pairs insertion. The throughput is increased by 33%, while the time overhead and average delay are reduced by 8% and 20%, respectively. Meanwhile, compared with Redis, PMEKV-FS has lower insert throughput, where the throughput is reduced by 26%, and the time overhead and average delay are increased by 6% and 32%, respectively. This further shows that the embedded key–value storage engine in NVM devices is an important factor in improving the key–value insert speed. Compared to PMEKV-M, the insert performance of PMEKV-NVM decreased slightly, and throughput is reduced by 1%, time overhead and average delay are increased by 3% and 7%, respectively. This is because the read and write speed of NVM devices is lower than DRAM.

Based on the testing result in Table 1, we change the number of threads to 2, 4, 8, and 16, respectively and test the throughput of key–value pairs insertion with multi-threads. The results are shown in Table 2.

The results in Table 2 show that PMEKV-NVM can improve the throughput of key–value pairs insertion with multi-threads compared with PMEKV-M, PMEKV-FS, Redis, MongoDB, Memcached, PMEM-KV and PMEM-Redis. Similar to the single-thread testing results, the improvement of PMEKV-NVM is still the largest compared to MongoDB and followed by Memcached. When the number of threads increases, the throughput of PMEKV-M, PMEKV-NVM, PMEKV-FS, Redis, PMEM-KV, and PMEM-Redis first increases and then decreases. The throughput reaches a peak when the number of threads is 8. When the number of threads increases from 8 to 16, the throughput of PMEKV-NVM, PMEM-Redis and Redis decreases by about 15%, PMEKV-M by 13%, and PMEKV-FS reached 22%. This indicates that PMEKV-NVM can better support concurrent access with multi-threads; it also shows that the conflict among several threads will become the factor affecting the insert performance of key–value store for the NVM device simulator. Unlike the results with single-thread, the throughput of PMEKV-NVM is slightly lower than that of Redis when the number of threads is 2, approximately 3%. However, the throughput of PMEKV-NVM is always higher than Redis after the number of threads increase. The gap of throughput between PMEKV-NVM and Redis is 3% when the number of threads is 8, which also indicates that PMEKV-NVM is favorable for multi-threaded key–value insertion. The throughput of PMEKV-FS is lower than PMEKV-NVM and Redis, which ranges from 38% to 54% and 36% to 52%, respectively. These results indicate that PMEKV-NVM can effectively improve the performance of the key–value pair insertion by reducing the number of reading and writing NVM devices.

### 5.3. Change the Number of Threads

We change the number of workload threads in YCSB named Workloada to test the throughput of the prototype. The thread number is 1, 2, 4, 8, 16, and the number of key–value pairs and access requests are 1000. The results are shown in Table 3.

There are many update operations in Workloada, with the ratio of read and write requests is 50%. It focuses on testing the update performance of key–value stores. From Table 3, it can be found that PMEKV-NVM always has a higher throughput than other prototypes on Workloada. The results also verify that PMEKV-NVM can improve write throughput by 3~16% and 1~8% compared with PMEM-KV and PMEM-Redis. Meanwhile, compared with PMEKV-FS, Redis, MongoDB and Memcached, the throughput of PMEKV-NVM is increased by 13~22%, 2~14%, 3.1 times to 3.9 times and 2.3 times to 3.4 times, respectively. When the number of threads is increased, the throughput of the prototypes is gradually increased before the number of threads exceeds 8 except for Memcached. When the number of threads increases from 8 to 16, the throughput of PMEKV-NVM and PMEKV-FS decreases by approximately 5%. The throughput gap of other prototypes is higher than the PMEKV-NVM and PMEKV-FS. PMEKV-M and Redis have the highest performance degradation by 15%. The throughput of Memcached begins to decrease after the number of threads exceeds 4. These results indicate that PMEKV-NVM has better adaptability for key–value pairs access by multi-thread.

Then, another workload in YCSB named Workloadb is used to test the throughput. We keep the testing configuration the same as the above. The results are shown in Table 4.

Unlike Workloada, Workloadb contains more read requests of key–value pairs. The ratio of read and write requests is 95/5. It mainly uses to test the read performance of key–value stores. As shown in Table 4, PMEKV-NVM always has a higher throughput than other prototypes. Meanwhile, PMEKV-NVM can increase more throughput on Workloadb than on Workloada. Compared to PMEM-KV and PMEM-Redis, the throughput of PMEKV-NVM has increased by 7~15% and 4~8%. It increases throughputs by 25~41%, 20~53%, 3.1 times to 5.2 times, and 3.5 times to 7.2 times, respectively, compared with PMEKV-FS, Redis, MongoDB, and Memcached. In addition, the throughput of PMEKV-FS exceeds Redis by 17% when the number of threads is 2. It is because the read speed of key–value stores is higher than the write speed (see results in Table 3 and Table 4). Thus it enhances the advantage of PMEKV-NVM relative to other prototypes. Unlike Workloada, the throughput decline occurs earlier on Workloadb with increased threads. The throughput begins to decrease when the number of threads exceeds 4. In particular, the throughput of PMEKV-FS starts to decrease after the number of threads exceeds 2. This is because the read throughput of key–value stores is higher than the write throughput, and the NVM device achieves I/O performance saturation much faster. These results also indicate that it is more important to optimize the read operation than write operation for key–value stores.

### 5.4. Changing the Number of Key–Value Pairs

**Figure 6:**
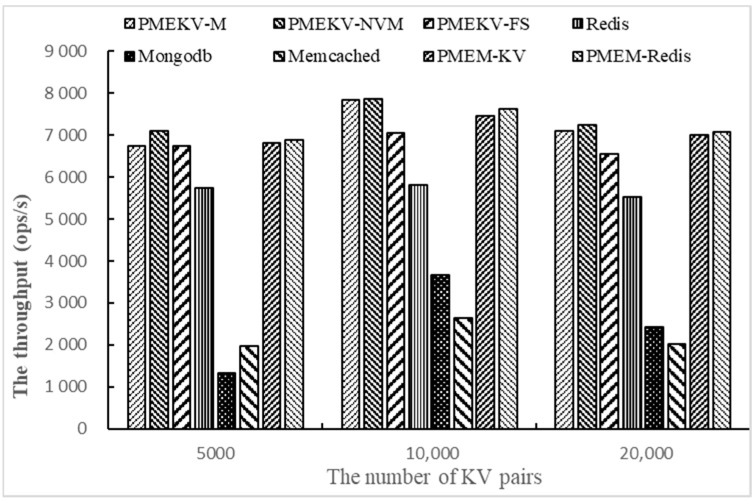
The throughput of changing the number of key–value pairs on Workloada.

**Figure 7:**
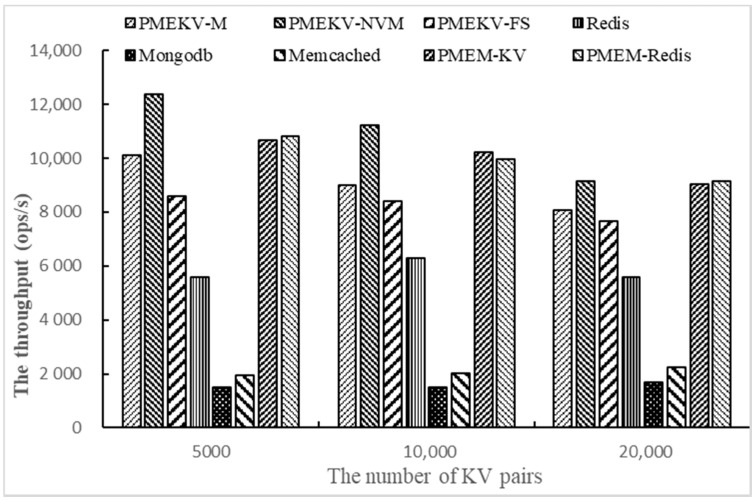
The throughput of changing the number of key–value pairs on Workloadb.

Then we test the throughput on Workloada and Workloadb with YCSB by changing the number of key–value pairs. The number of key–value pairs is set to 5000, 10,000 and 20,000, respectively. We only use one thread and 1000 access requests. The results are shown in Figure 6 and Figure 7.

The purpose of changing the number of key–value pairs is to test the adaptability of the prototypes. Figure 6 gives the results on Workloada, which shows PMEKV-NVM always has a higher throughput than other prototypes. When the number of key–value pairs increases from 5000 to 20,000, its throughput is increased by 5~11%, 23~35%, 1.1 times to 4.3 times and 1.9 times to 2.5 times, respectively, compared with PMEKV-FS, Redis, MongoDB and Memcached. These results indicate that PMEKV-NVM has higher write throughput with a large number of key–value pairs. Meanwhile, its throughput is increased by 3~6% and 3% compared with PMEM-KV and PMEM-Redis. The throughput of PMEKV-FS is higher than Redis. These results show that the separation of the key and value for key–value pairs can reduce the size of index and the device memory consumption in NVM devices, thus improve the management efficiency with a large number of key–value pairs.

Figure 7 shows the results of Workloadb. When the number of key–value pairs increases from 5000 to 20,000, the throughput of PMEKV-NVM is increased by 20~44%, 64%~1.2 times, 4.5 times~7.3 times and 3.1, respectively, compared with PMEKV-FS, Redis, MongoDB and Memcached. Moreover, it is increased by 1~16% and 0.3~14%, compared with PMEM-KV and PMEM-Redis. Meanwhile, the throughput of PMEKV-FS is also higher than Redis. These results show that the separation of the key and value can reduce the size of the index and the device memory consumption in NVM devices, thus improve the management efficiency with a large number of key–value pairs.

### 5.5. Changing the Number of Access Requests

**Figure 8:**
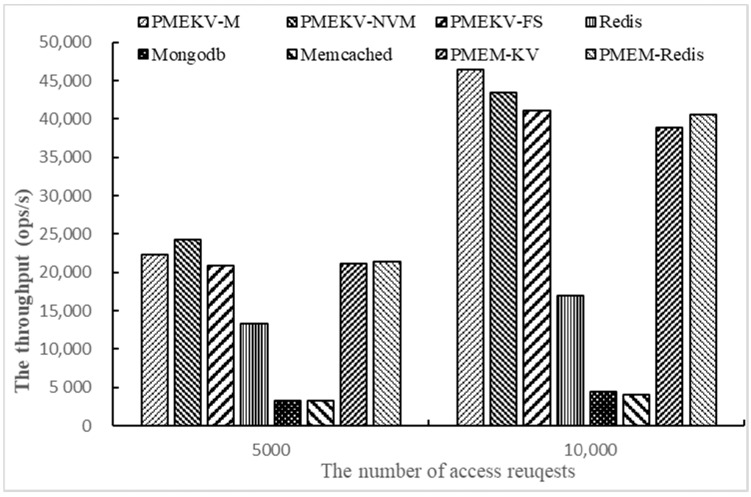
The throughput of changing the number of access requests on Workloada.

**Figure 9:**
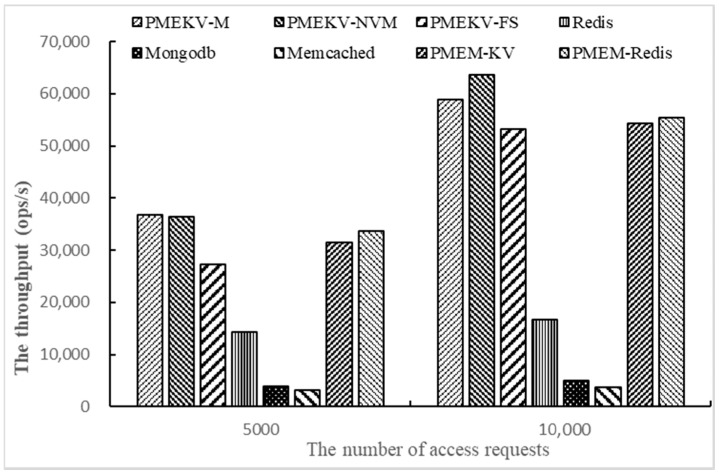
The throughput of changing the number of access requests on Workloadb.

Finally, we test the throughput on Workloada and Workloadb with YCSB by changing the number of access requests. The number of access requests is 5000 and 10,000, and one thread and 1000 key–value pairs are used here. The results are shown in Figure 8 and Figure 9.

The results in Figure 8 and Figure 9 are similar to the results in 5.4. PMEKV-NVM has the highest throughput on Workloada and Workloadb. Meanwhile, the throughput of all prototypes on Workloadb is higher than Workloada, and this denotes that the prototype deals with read operations faster than write operations. When the number of access requests increases from 5000 to 10,000, the throughput of PMEKV-NVM and PMEKV-FS increase by 80% and 97%, respectively, on Workloada, and by 75% and 95%, respectively on Workloadb. The increased ratio of throughput is close to the requests. It means that PMEKV-NVM and PMEKV-FS have strong adaptability for the variation of the number of access requests. At the same time, the throughput of Redis and Memcached are just increased by 37% and 21%, respectively, on Workloada, and are just increased by 17% and 25%, respectively, on Workloadb. The increased ratio of throughput for Redis and Memcached is slower than the access requests. In addition, the throughput of PMEM-KV and PMEM-Redis increase by 84% and 89%, respectively, on Workloada, and increase by 73% and 65% on Workloadb. The increased ratio of throughput for Redis and Memcached is also slower than PMEKV-NVM and PMEKV-FS. This also proves that the key and value separation used in PMEKV-NVM and PMEKV-FS can effectively reduce the size of the index and thus have better adaptability.

At the same time, corresponding to the results in Section 5.3, it can be found that multi-threads more affect the key–value stores throughput than access requests. Therefore, how to improve the index concurrency is an important issue. In addition, comparing the results in Figure 8 and Figure 9, we can see the throughput changes are generally more obvious on Workloada than on Workloadb. This is because there are more read operations in Workloadb, and its original throughput is higher; thus, the growth of throughput is slightly lower. At the same time, the gap of the increase ratio of throughput is only about 5% on Workloada and Workloadb for PMEKV-NVM and PMEKV-FS when the request increases from 5000 to 10,000, which also shows that PMEKV-NVM and PMEKV-FS have better adaptability for different workloads.

## 6. Conclusions and Future Works

We design the new embedded key–value store for the NVM device simulator. The embedded processor in NVM devices is used to manage the key–value pairs, and thus, the data transfer between NVM devices and main memory can be reduced to improve the I/O performance of key–value store. Meanwhile, the I/O software stack for key–value store is shortened to adapt to the characteristic of the NVM device. We implement the prototype based on PMEM and compare it with several key–value stores. The results show that PMEKV has the advantage of throughput and adaptability over current key–value stores.

Now we just use the B+ tree to index the key of key–value pairs; it still has efficiency and concurrency limitations. It is an important reason that the throughput of PMEKV-FS is lower than PMEM-Redis and PMEM-KV, and it also affects the throughput of PMEKV-NVM. In the future, we will study how to design new index strategies.

## Figures and Tables

**Table 1:** The results of key–value pairs insertion.

	Throughput (ops/s)	Run Time (ms)	AVL (ms)
PMEKV-M	4830	207	164
PMEKV-NVM	4765	215	176
PMEKV-FS	3571	235	221
Redis	4500	222	150
MongoDB	1364	733	503
Memcached	1818	550	417
PMEM-KV	4539	220	148
PMEM-Redis	4603	218	144

**Table 2:** The throughput of key–value pairs insert with multi-threads.

Throughput (ops/s)	2 Threads	4 Threads	8 Threads	16 Threads
PMEKV-M	5586	6015	6517	5789
PMEKV-NVM	5451	6113	7143	6172
PMEKV-FS	3728	4452	4912	4018
Redis	5649	6037	6944	6012
MongoDB	1808	2409	2493	2770
Memcached	1960	2481	2174	2247
PMEM-KV	5397	6064	6915	5873
PMEM-Redis	5702	6057	6995	6112

**Table 3:** The results of changing the number of threads on Workloada.

Throughput (ops/s)	1 Thread	2 Threads	4 Threads	8 Threads	16 Threads
PMEKV-M	8196	8771	9345	10,989	9528
PMEKV-NVM	7863	9233	9708	11,069	10,526
PMEKV-FS	6992	7756	8564	9046	8633
Redis	5714	8124	9618	10,894	9523
MongoDB	1262	1893	2183	2717	2409
Memcached	1890	2145	2906	2597	2369
PMEM-KV	7455	8530	9434	9503	9148
PMEM-Redis	7514	8823	9673	11,014	9749

**Table 4:** The results of changing the number of threads on Workloadb.

Throughput (ops/s)	1 Thread	2 Threads	4 Threads	8 Threads	16 Threads
PMEKV-M	11,928	12,522	12,904	11,869	10,204
PMEKV-NVM	10,869	12,157	12,987	11,363	10,869
PMEKV-FS	8345	9539	9185	8923	8725
Redis	6451	7936	9708	9433	8547
MongoDB	1615	1965	2403	2439	2652
Memcached	1700	1488	2695	2525	2004
PMEM-KV	10,154	10,538	11,293	10,481	9588
PMEM-Redis	10,358	11,254	12,036	10,879	10,258
